# Puparial Cases as Toxicological Indicators: Bioaccumulation of Cadmium and Thallium in the Forensically Important Blowfly *Lucilia sericata*

**DOI:** 10.3389/fchem.2020.586067

**Published:** 2020-11-17

**Authors:** Julita Malejko, Krzysztof Deoniziak, Marlena Tomczuk, Joanna Długokencka, Beata Godlewska-Żyłkiewicz

**Affiliations:** ^1^Department of Analytical Chemistry, Faculty of Chemistry, University of Bialystok, Białystok, Poland; ^2^Laboratory of Insect Evolutionary Biology and Ecology, Faculty of Biology, University of Bialystok, Białystok, Poland

**Keywords:** entomotoxicology, *Lucilia sericata*, cadmium, thallium, bioaccumulation factor, ICP-MS

## Abstract

In this study, we present entomotoxicological data on the accumulation of cadmium and thallium in a forensically important blowfly, *Lucilia sericata*, and evaluate the reliability and utility of such information as toxicological evidence for poisoning as a cause of death. We observed that Cd and Tl content in different growing stages of *L. sericata* (larvae, puparial cases, and adults) was increasing with increasing metal concentration in the feeding substrate, namely metal-enriched liver. However, patterns of accumulation differed between the two metals investigated, showing a linear relationship for Cd and a saturable pattern for Tl. For cadmium, the highest bioaccumulation factor (BAF) was found in the larval stage (in the range of 0.20–0.25), while for thallium, puparial cases accumulated more metal than the other stages tested (BAF in the range of 0.24–0.42). Thallium was also observed to have a negative effect on larval growth, resulting in lower weight and smaller puparial size. With this study, we update the information on the bioaccumulation of cadmium in forensically important blowflies and provide the first report on the bioaccumulation of thallium as well as its developmental impact in blowflies. Specifically, our results suggest that analysis of puparial cases could yield useful information for entomotoxicological investigations. The content of Cd and Tl in larvae, puparial cases, and adults of *L. sericata* was determined by inductively coupled plasma mass spectrometry (ICP-MS). The validation parameters of the method such as sensitivity, detection limits, quantification limits, precision, and accuracy were evaluated. The method detection limit (MDL) for all types of samples was in the range of 1.6–3.4 ng g^−1^ for Cd and 0.034–0.15 ng g^−1^ for Tl, and the accuracy of the method was confirmed by a high recovery of metals from certified reference materials (91.3% for Cd and 94.3% for Tl).

## Introduction

Forensic entomology is commonly associated with death investigations, in which its main application is in determination of the minimum postmortem interval (_min_PMI). This is accomplished by estimating the time of insect colonization, based on knowledge of the rate of development of pioneer colonizers and on the succession of different insect species during the decomposition of animal/human remains (Greenberg, 1991; Campobasso et al., 2004; Bugelli et al., 2017a). Entomological evidence can also be used to support investigators in determining the presence of drugs or toxins in the body at the time of death, even if decomposition is at an advanced stage.

The use of insects as a source of toxicological evidence, known as entomotoxicology, relies on the fact that many insects can accumulate pollutants from their feeding substrates in their tissues (Pounder, 1991). Of the many species that may be present on or in a dead body, blowflies (Diptera: Calliphoridae) are usually the first to appear on the crime scene, and they may provide crucial information—such as the cause of death—months or even years after body decomposition (Wang et al., 2008; Byrd and Castner, 2010; Braga et al., 2013). This approach was first used in 1980, when phenobarbital was detected in fly larvae collected from a skeletonized body. This finding fundamentally altered the determination of the cause of death, and gave rise to a new application for entomology in forensic science (Beyer et al., 1980). Since then, blowflies have been used as bioindicators of toxins present in bodies that are in an advanced stage of decomposition and thus lack direct forensic matrices (e.g., blood, organs) (Greenberg, 1991; Campobasso et al., 2004; Bugelli et al., 2017a). In a similar way, fly larvae may be also used as indicators of environmental pollution (Owings et al., 2019). To date, various drugs and toxic substances have been detected in different developmental stages of blowflies, in both experimental studies on animal carcasses and human bodies as well as in bodies analyzed in criminal investigations (reviewed in Silva et al., 2017). Entomotoxicological studies focus not only on the qualitative and quantitative analysis of toxicants, but also on their impact on insect growth, development, and survival, which enable an accurate determination of PMI. Although there have been some critiques of the use of entomotoxicology in forensic science (Gaudry et al., 2001; Tracqui et al., 2004), many studies have shown that insects can provide reliable evidence in toxicological analysis during criminal investigations (Silva et al., 2017; Chophi et al., 2019).

Cadmium (Cd) and thallium (Tl) are toxic metals that are responsible for many accidental and intentional poisonings (Cavanagh, 1991; Desenclos et al., 1992; Meggs et al., 1994; Hoffman and Hoffman, 2000; Chang et al., 2012; Nishijo et al., 2017; Rafati Rahimzadeh et al., 2017). Of the two, thallium is more toxic to humans, with a lethal dose between 6 and 40 mg kg^−1^, compared to a lethal dose of more than 70 mg kg^−1^ for cadmium (Agency for Toxic Substances Disease Registry, 1992, 1999). Cadmium accumulates in almost all human tissues and induces production of metallothionein, a cysteine-rich metal-binding protein. Its toxic effect is observed in the liver, kidneys, skeletal system, and cardiovascular system, and it has also been classified as a human carcinogen. The biological half-life of Cd in the human body has been estimated to be 10–30 years (Sigel et al., 2013; Powers and Dean, 2015; Lech and Sadlik, 2017). Monovalent thallium is similar to potassium in ionic radius and electrical charge, which contributes to its toxic nature. It interferes with many enzymes and has a strong affinity to –SH groups. In the past, thallium salts were widely used as rodenticides, but these were withdrawn from the market due to its high toxicity and the large number of human poisonings, accidental or intentional. Gastroenteritis, polyneuropathy, and alopecia are considered the classic syndromes of thallium poisoning (Reith, 2009; Karbowska, 2016).

With this in mind, we decided to perform a quantitative analysis of Cd and Tl accumulation in a forensically important blowfly in order to determine if such information could be useful as toxicological evidence for poisoning as a cause of death. To date, the effect of cadmium on forensically important blowflies (Diptera: Calliphoridae) has been investigated mainly in the context of life history traits (e.g., survival or development rate) in the species *Chrysomya albiceps* (Al-Misned, 2001, 2003), *Chrysomya megacephala* (Singh and Bhupinderjit, 2017), and *Lucilia sericata* (Simkiss et al., 1993; Moe et al., 2001); of these studies, only the latter two conducted experiments to assess the content of cadmium in insects. Instead, the only published study on the effects of thallium in insects characterized the tolerance of *Chironomus riparius* (Diptera: Chironomidae) to waterborne exposure (Belowitz et al., 2014). To the best of our knowledge, thallium accumulation in blowflies, and the potential value of such data for the field of entomotoxicology, has not yet been studied.

As the model species in our study we used the cosmopolitan and necrophagous fly *L. sericata* (Meigen, 1826) (Diptera: Calliphoridae), which occurs in a broad range of ecosystems throughout the world. *L. sericata* is a common model organism in forensic studies (Byrd and Castner, 2010). As mentioned above, adult flies of this species are often the first insects to arrive at a corpse, and larvae are used in estimation of PMI (Reibe et al., 2010). *L. sericata* has been widely used in experimental studies of entomotoxicology (reviewed in Silva et al., 2017), variation in life history traits (Gallagher et al., 2010), or alterations in arthropod communities and succession patterns on cadavers (Prado e Castro et al., 2012). Here, we characterized the accumulation of cadmium and thallium in different developmental stages of *L. sericata* and assessed the impact this exposure had on insect growth (weight of third-instar larvae, length and width of puparia). In addition to the larval and adult stages, which are typically used in entomotoxicological studies (Chophi et al., 2019), we also chose to analyze empty puparial cases. Puparial cases are shed by emerging adults, and may be present near cadavers up to several years after death (Braga et al., 2013). Cases are formed during pupariation from the cuticle of third-instar larvae and can serve as an alternative material for toxicological analysis when living insects and suitable tissues are missing. Puparial cases have been shown to be useful in determination of PMI (Zhu et al., 2017) and in the identification of species present on a cadaver (Braga et al., 2013). They can also provide important evidence during toxicological analysis, as drugs such as cocaine (Nolte et al., 1992), amitriptyline (Miller et al., 1994), and metabolites of methylenedioxyamphetamine (Goff et al., 1997) have been detected in puparial cases. However, there have only been a few analyses of toxic metals in puparial cases (Silva et al., 2017; Chophi et al., 2019). Interestingly, an earlier study on *L. sericata* suggested a mechanism for detoxification via puparial cases (Simkiss et al., 1993), a hypothesis that we test further in the current work.

Cadmium and thallium content was determined in various developmental stages of *L. sericata* using inductively coupled plasma mass spectrometry (ICP-MS). ICP-MS is one of the most widely used analytical techniques in inorganic testing laboratories, due to its wide dynamic range, high sensitivity, low limits of detection, and fast multi-elemental analysis (Sakai, 2015). Data quality can be improved significantly through the use of an octopole-based collision/reaction cell operating in helium collision mode, which is very effective in eliminating a wide range of matrix-based interference. In this work, we also describe a methodological protocol to help other researchers to achieve comparable results.

## Materials and Methods

### Instrumentation

An Agilent 8800 Triple Quadrupole ICP-MS (ICP-QQQ, Agilent Technologies, Singapore) was used to determine the Cd and Tl content of biological samples. The apparatus was equipped with a MicroMist glass concentric nebulizer, a Peltier-cooled quartz double-pass Scott-type spray chamber, a quartz torch with a 2.5-mm i.d., Ni interface cones, an x-lens, and an octopole reaction system (ORS3). Samples were introduced directly into the ICP-MS using a SPS 4 autosampler with a standard peristaltic pump and tubing (i.d. 1.02 mm).

A Solaar M6 (Thermo Electron Corporation, Gloucester, UK) atomic absorption spectrometer, equipped with an electrothermal atomizer and a Zeeman background correction system, was used to determine the Cd concentration of liver samples.

A Milestone ETHOS Plus Microwave Labstation (Italy) was used for microwave-assisted digestion of samples. Samples were homogenized using a blender with a glass jar (Łucznik, Poland), a mechanical micro-stirrer (MPW-321; Mechanika Precyzyjna, Poland), and a SONOREX DIGIPLUS ultrasonic bath (DL 255H; ultrasonic nominal power: 160 W, power settings: 20–100%, ultrasonic frequency: 35 kHz) from Bandelin (Germany). A Memmert UE 400 oven (Germany) was used to dry biological samples. A DSX110 inverted microscope (Olympus Corporation, Japan) was used for measurements of puparial length and width.

### Reagents and Materials

All solutions were prepared in Milli-Q water, which was obtained from a Millipore Direct-Q 3 UV water purification system (France). Concentrated HNO_3_ (≥69%, TraceSelect, Fluka, France) and H_2_O_2_ (≥30%, for trace analysis, Sigma-Aldrich, France) were used for sample digestion. Indium (1,000 mg L^−1^, In(NO_3_)_3_ in 2% HNO_3_), supplied by Merck (Germany), was used as an internal standard for ICP-MS measurements. The ICP-MS stock tuning solution contained 10 mg L^−1^ each of Li, Y, Ce, Tl, and Co in a matrix of 2% HNO_3_, and was supplied by Agilent Technologies (USA). The cadmium standard solution (1,000 mg L^−1^ Cd in 0.5 mol L^−1^ HNO_3_, traceable to SRM from NIST, Certipur®) and thallium ICP standard (1,000 mg L^−1^ Tl in 2–3% HNO_3_, traceable to SRM from NIST, Certipur®) were obtained from Merck (Germany).

Two certified reference materials (CRMs) were used to check the accuracy of the method: MODAS-4 Cormorant Tissue (MODAS, Poland), with a certified Cd concentration of 17.2 ± 2.1 ng g^−1^, and Seronorm? Trace Elements Urine L-2 (SERO, Norway), with a certified Tl concentration of 9.70 ± 0.67 μg L^−1^ (acceptable range: 8.36–11.04 μg L^−1^).

### Substrate Preparation

Fresh homogenized pig liver was enriched with Cd or Tl and used as a substrate for larvae of *L. sericata*. The pig liver used in both cases was acquired from a single individual. Each portion of liver (100 g) was placed in glass container, to which was added 20 mL standard solution of either Cd (C_1_: 0.3254 mg; C_2_: 0.6538 mg; C_3_: 1.2832 mg) or Tl (T_1_: 0.0755 mg; T_2_: 0.3268 mg; T_3_: 0.6542 mg), prepared with Milli-Q ultrapure water. Control samples (C_0_ and T_0_) were prepared by mixing 10 mL of Milli-Q water with 100 g of fresh homogenized pig liver. Each sample was sonicated in an ultrasonic bath for 30 min at 60% power. The vessels were cooled with ice to avoid heating during the sonication process. Next, from each of the prepared substrates (C_0−3_ and T_0−3_), three portions (20 g each) were taken and transferred to 250 mL plastic containers. Summarizing, three individual samples of each liver substrate (control or with one of the three concentrations of metal) were prepared. After preparation, 5 g of each substrate was used to determine its thallium or cadmium concentration. These samples were stored at −20°C until analysis.

Rigorous precautions were taken to avoid contamination throughout the procedure. Prior to use, all plastic materials and glassware were thoroughly rinsed with Milli-Q water, soaked for 48 h in 2 mol L^−1^ nitric acid, and finally rinsed several times with Milli-Q water.

### Breeding and Sampling

First-instar larvae of *L. sericata* were provided by Biomantis Ltd., Kraków, Poland. Newly hatched larvae were packed in 100-mL plastic containers with an addition of saline, and shipped as a medical consignment to the Faculty of Biology, University of Bialystok, Poland. The consignment was protected by thermo-insulating packaging with the addition of a cooling insert. The package was delivered within 24 h of shipment. After delivery, the plastic container was removed from the packaging in order to acclimatize the larvae to room temperature (21.5°C). After 1 h, larvae were deposited on the substrate in random order.

In general, 47 larvae were placed in each 250-mL plastic container (with either control substrate or substrate enriched with different concentrations of cadmium or thallium), which was then covered with sterile gauze. There were three replicates for each treatment. All containers were then placed in one clear plastic box (58.3 × 39.2 × 18.3 cm), together with two 250-mL plastic containers half-filled with distilled water (to maintain humidity) and a digital weather station for measuring temperature and humidity during the experiment. The container was covered with a clear plastic lid in a way that allowed air exchange. Larvae were reared in an air-conditioned room at 21.5°C and 80–90% humidity, with 10 h daylight and 14 h darkness. Larvae were checked twice a day.

Third-instar larvae (hereafter “larvae”) were moved to a new 250-mL plastic container after about 96 h, when they entered their wandering stage and started to move intensively on the sides of the container and the sterile gauze. Before being placed in their new container, all larvae were rinsed with Milli-Q water and dried on blotting paper. In line with the rules for the collection of insects from the scene (Brundage and Byrd, 2016), a sample of 15 larvae was collected from each of the cadmium (C_0−3_) and thallium (T_0−3_) substrates; each sample was then divided into three subsamples, which were weighed and stored at −20°C until further analysis. To avoid including any changes in larval weight that occurred during storage, weights were recorded immediately after collection (Bugelli et al., 2017b). The remaining larvae in their plastic containers were then placed back in the clear plastic box for pupation. After all larvae entered the pupal stage, the puparia from the Tl-treated groups were measured with a DSX110 inverted microscope. Adults started to emerge 17 days after entering the wandering stage. After emergence, samples of puparial cases and adults were collected and stored at −20°C for further analysis. In total, for each substrate (control or different Cd/Tl concentration), nine sub-samples of larvae, puparial cases, and adults were collected. The experiments were conducted in accordance with Polish law (e.g., Animal Protection Law, Animal Husbandry Act) and all provisions and regulations stipulated therein.

### Pre-treatment of Samples and CRMs

Biological samples were dried in an oven for 12 h until they reached a constant weight (liver samples at 105°C; samples of larvae, puparial cases, and adults of *L. sericata* at 40°C). Dry samples of liver (0.2–0.3 g), larvae (0.06–0.2 g), puparial cases (0.01–0.02 g), and adults (0.05–0.1 g), as well as samples of the CRM MODAS-4 Cormorant Tissue (0.2 g) were placed in Teflon vessels for microwave-assisted acid digestion. Concentrated HNO_3_ (2 mL) and H_2_O_2_ (1 mL) were added, and the vessels were left for 1 h for sample pre-digestion. Next, the vessels were sealed and heated in the microwave system. The digestion conditions were: 1 min at 150°C, 1 min at 160°C, 5 min at 200°C, 5 min at 210°C, and 5 min at 210°C. For the Seronorm? Trace Elements Urine L-2 CRM, a sample of freeze-dried material was reconstituted in 5 mL of Milli-Q water, then 1 mL of this solution was placed in a quartz crucible and digested in hot concentrated HNO_3_ (0.5 mL) and H_2_O_2_ (0.5 mL). After cooling, all digests were transferred to polyethylene vessels and stored in a refrigerator. Samples were diluted with Milli-Q water or 2% HNO_3_ prior to analysis.

### Conditions for the Measurement of Cd and Tl

The optimized ICP-MS operating conditions for the determination of Cd and Tl in mineralized samples are given in Supplementary Table 1. The monitored isotopes were ^111^Cd, ^114^Cd, and ^205^Tl. These were monitored both in standard mode (no gas) and in He mode (using helium as a collision gas with a flow rate of 5 mL min^−1^). All calibration standards were prepared in 2% HNO_3_. Indium (^115^In) was used as an internal standard. Electrothermal atomic absorption spectrometry (ETAAS) was used to determine Cd concentrations in liver samples during optimisation of substrate preparation. The cadmium hollow cathode lamp (Thermo Scientific, UK) was operated at 5 mA current. All measurements were performed using standard pyrolytically coated graphite furnaces. Absorbance signals were measured with 0.5 nm spectral bandpass at 228.8 nm. The time/temperature program was: drying at 100°C for 30 s, 800°C for 20 s, ashing at 1,000°C for 3 s, and atomization at 2,500°C for 3 s. Magnesium nitrate (20 μg) was used as a chemical modifier for the determination of Cd concentration. The concentration of each analyte was measured from the calibration graph.

### Calculation of Bioaccumulation Factor

The bioaccumulation factor (BAF) was calculated as the ratio of the metal content in tested samples of *L. sericata* to the metal content in the feeding substrate, as follows:

$$\begin{array}{l} BAF=\frac{\text{metal content in tested organism }\left(\text{μg }{\text{g}}^{-1}\text{ d}.\text{w}.\right)}{\text{metal content in feeding substrate }\left(\text{μg }{\text{g}}^{-1}\text{ d}.\text{w}.\right)} \end{array}$$

### Statistical Analysis

The Mann–Whitney *U*-test was used to evaluate differences in Cd and Tl concentration and in the bioaccumulation factors of the three tested developmental stages of *L. sericata*. Monte Carlo permutations, with 10,000 randomizations and a 99% confidence interval, were used to test for statistical significance. The weight of larvae and the length and width of puparia were compared among groups using one-way ANOVA and Tukey HSD *post-hoc* tests. The distribution of the data was analyzed with a Shapiro–Wilk test. All of the statistical analyses were two-tailed and were performed using SPSS Statistics v. 26 (IBM Corporation, USA).

## Results

### Optimization of the Method of Substrate Preparation

We first conducted preliminary studies to optimize the method of preparation of the metal-enriched liver substrate and to ensure that the metal was distributed homogenously throughout the entire matrix. The liver (600 g) was rinsed with distilled water, quartered, and blended for 10 min. Liver samples (100 g) were then homogenized with different volumes of cadmium solution (10 or 20 mL, each containing 0.325 mg Cd). Half of the samples were subjected to mechanical stirring (for 5 min) and the other half to sonication in an ultrasonic bath (for 30 min, at 60% power). During sonication, the vessels were cooled with ice to avoid heating. After homogenization, four randomly selected sub-samples (~1 g each) were dried in an oven for 12 h at 105°C, then digested in a microwave digestion system according to the procedure described in Section Pre-treatment of samples and CRMs. The Cd content of liver samples was determined by ETAAS under the conditions described in Section Conditions for the measurement of Cd and Tl. The instrumental detection limit (IDL), calculated as three times the standard deviation of the response for a blank sample (2% HNO_3_) divided by the slope of the calibration curve, was 0.15 ng mL^−1^. The concentration of Cd in control liver tissue was 0.066 ± 0.010 μg g^−1^ (d.w.).

In total, there were four groups of enriched liver samples, which differed in the volume of Cd solution added during preparation (10 or 20 mL, both containing 0.325 mg Cd) and method of homogenization (mechanical stirring or ultrasound); four samples were analyzed from each group. The average concentration of Cd in tissue homogenized by mechanical stirring was 13.99 ± 0.56 μg g^−1^ in the 10-mL group and 13.04 ± 0.56 μg g^−1^ in the 20-mL group. When tissues were homogenized by ultrasound, the average Cd concentration was 12.41 ± 0.97 μg g^−1^ in the 10-mL group and 12.91 ± 0.35 μg g^−1^ in the 20-mL group. The best repeatability of measurement (relative standard deviation, or RSD, of 2.7%), and thus better homogeneity of the liver sample, was therefore achieved using ultrasonic homogenization after the addition of 20 mL of cadmium solution. These conditions were used to prepare liver substrate for all further experiments. Moreover, this homogenization method enabled the simultaneous preparation of multiple samples and avoided loss or contamination via laboratory tools during substrate preparation.

### Determination of Cd and Tl by ICP-MS: Validation of the Method

Calibration graphs were recorded for two cadmium isotopes (^111^Cd and ^114^Cd) and one thallium isotope (^205^Tl) with and without the use of collision cell technology. The signal of the internal standard was more stable in He mode. However, the results were comparable in both modes, indicating selectivity of the method and a lack of interference in the tested samples. For the calculation of results, the signals registered in both measurement modes were used. Intermediate precision, expressed as the RSD of the slopes of calibration graphs recorded on different days, was in the range of 0.8–4.1% (*n* = 3) for cadmium and 3.2–6.7% (*n* = 4) for thallium. The instrumental detection limit (IDL) calculated as three times the standard deviation (SD) of the signal of blank sample (SD_blank_) (2% HNO_3_) divided by the slope of the calibration graph (a), was 0.049 ng g^−1^ for Cd and 0.015 ng g^−1^ for Tl, while the instrumental quantification limit (IQL) calculated as (10 × SD_blank_)/a was 0.16 ng g^−1^ for Cd and 0.051 ng g^−1^ for Tl. The method detection limit (MDL), calculated as 3 × SD_control_ of the response of control samples (mineralized samples of liver and different developmental stages of *L. sericata*) divided by the slope of the calibration graph (a), and the method quantification limit (MQL), calculated as (10 × SD_control_)/a, are presented in Table 1. These values are inherent to the chemical measurement process and represent both instrumental and procedural components, i.e., dilution and mineralization. These low MDL and MQL thus enabled the detection of cadmium and thallium in as little as 0.01 g of sample for puparial cases. The repeatability of results (as RSD in %) was calculated for three sub-samples of liver enriched with each concentration of metal. It was in the range of 0.24–2.3% for Cd and 1.1–6.8% for Tl. The reproducibility of the results (RSD) was evaluated using the three sub-samples taken from each treated group of larvae, puparial cases, and adults, and was found to be in the range of 1.4–26.8% for cadmium and 2.5–25.1% for thallium. Only in two cases the RSD was found to be higher than 30% (34.1% for Cd and 31.4% for Tl). These higher RSD values may have resulted from the fact that, as the amount of material to be tested was sometimes very small, the samples were not homogenized before sampling (individual flies were mineralized).

**Table 1:** Method detection limit (MDL) and method quantification limit (MQL) of the ICP-MS method of Cd and Tl determination in biological samples.

	**Cadmium**		**Thallium**	
	**MDL (ng g^**−1**^)**	**MQL (ng g^**−1**^)**	**MDL (ng g^**−1**^)**	**MQL (ng g^**−1**^)**
Liver	1.6	5.2	0.036	0.12
Larvae	1.6	5.5	0.094	0.31
Puparial cases	2.2	7.3	0.15	0.51
Adults	3.4	11.4	0.034	0.11

The determined concentrations of metals in CRMs (15.7 ± 1.5 ng g^−1^ Cd in MODAS-4 Cormorant Tissue, 9.15 ± 0.23 μg L^−1^ Tl in Seronorm? Trace Elements Urine L-2) were consistent with the reference values, which verified the accuracy of the method. The average recoveries of analytes from the CRMs, with the estimated expanded uncertainties for *k* = 2, were 91.3 ± 12.6% for Cd and 94.3 ± 6.9% for Tl. The uncertainties were estimated using the single laboratory validation concept (Barwick and Ellison, 2000).

### Cadmium Accumulation in Larvae, Puparial Cases, and Adults of *L. sericata*

First, we used ICP-MS to determine the Cd content in control (unenriched) liver samples and liver tissues enriched with different concentrations of metal, which were all used as substrates for larvae. In control liver tissues (C_0_), the Cd content (as d.w.) was 94.1 ± 5.9 ng g^−1^, while in treated liver samples, Cd concentrations were much higher: 11.566 ± 0.028 μg g^−1^ in C_1_, 23.83 ± 0.54 μg g^−1^ in C_2_, and 46.98 ± 0.56 μg g^−1^ in C_3_. Substrate C_3_ contained a concentration of Cd that was equal to the median lethal dose (LD_50_: 50 μg g^−1^) for rats and mice (Agency for Toxic Substances Disease Registry, 1999).

**Figure 1:**
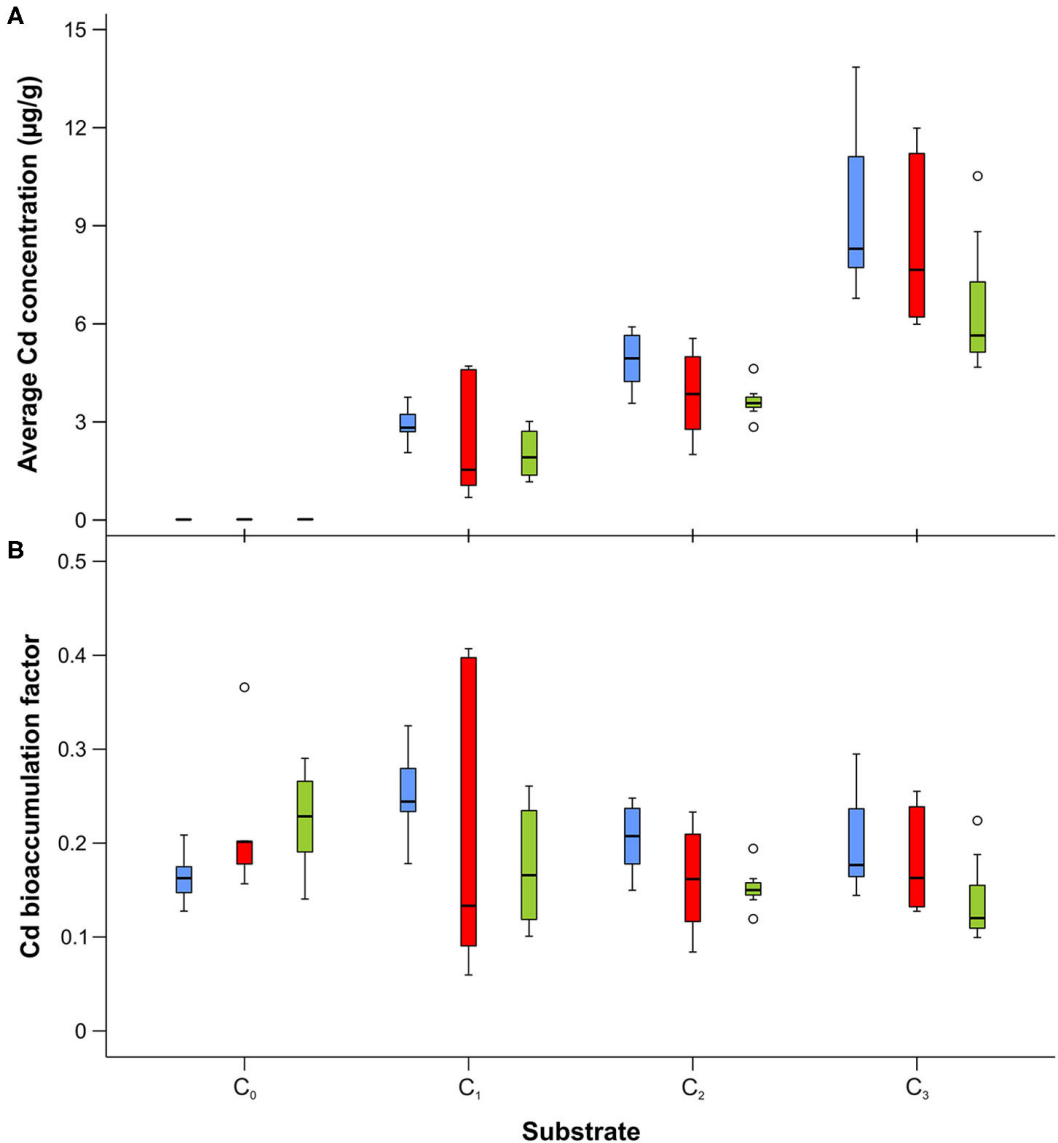
Box and whisker plots showing differences in average cadmium concentration **(A)** and bioaccumulation factor **(B)** in larvae (blue), puparial cases (red), and adults (green) of *Lucilia sericata* exposed to different Cd concentration (C_0_-C_3_). Boxes indicate median and first and third quartiles. Whiskers represent the minimal and maximal values within 1.5 times the interquartile range. Open circles are outliers with values more than 1.5 times the interquartile range.

Cadmium was detected in all developmental stages (larvae, adults, and puparial cases) of *L. sericata* collected from control and enriched substrates, with the mean cadmium concentration in each stage given in Supplementary Table 2. Concentrations in studied samples ranged from 15.4 ng g^−1^ to 9.5 μg g^−1^. Cadmium concentrations increased significantly in larvae, puparial cases, and adults with an increase in the Cd concentration in the food substrate (Figure 1, Table 2), and demonstrated a linear relationship in the studied concentration range (Supplementary Figure 1). The concentration of cadmium was significantly higher in larvae than in adult flies for all three Cd-treated groups (C_1_-C_3_), while it was higher in puparial cases than in adult flies for group C_3_ (highest substrate concentration) only (Figure 1, Table 3).

**Table 2:** Difference of Cd concentration determined in *Lucilia sericata* larvae, puparial cases, and adults from control and Cd treated groups (C_0_-C_3_).

**Sample**	**Larvae**			**Puparial cases**			**Adults**		
**Substrate**	**C_**0**_**	**C_**1**_**	**C_**2**_**	**C_**0**_**	**C_**1**_**	**C_**2**_**	**C_**0**_**	**C_**1**_**	**C_**2**_**
C_1_	*N* = 15	–	–	*N* = 14	–	–	*N* = 14	–	–
	*Z* = −3.182			*Z* = −3.130			*Z* = −3.130		
	***p<*** **0.001**			***p*** **=** **0.001**			***p*** **=** **0.001**		
C_2_	*N* = 15	*N* = 18	–	*N* = 14	*N* = 14	–	*N* = 15	*N* = 15	–
	*Z* = −3.182	*Z* = −3.400		*Z* = −3.130	*Z* = −1.214		*Z* = −3.240	*Z* = −3.125	
	***p<*** **0.001**	***p<*** **0.001**		***p*** **=** **0.001**	*p* = 0.263		***p<*** **0.001**	***p*** **=** **0.001**	
C_3_	*N* = 15	*N* = 15	*N* = 15	*N* = 15	*N* = 15	*N* = 15	*N* = 15	*N* = 15	*N* = 16
	*Z = –*3.182	*Z = –*3.576	*Z = –*3.576	*Z = –*3.240	*Z = –*3.240	*Z = –*3.240	*Z = –*3.240	*Z = –*3.240	*Z = –*3.361
	***p<*** **0.001**	***p<*** **0.001**	***p<*** **0.001**	***p<*** **0.001**	***p=*** **0.001**	***p<*** **0.001**	***p=*** **0.001**	***p=*** **0.001**	***p=*** **0.001**

*Bold values denote statistical significance at the p <0.05*.

**Table 3:** Difference of Cd concentration in *Lucilia sericata* larvae, puparial cases, and adults from groups treated with the same Cd concentration.

**Substrate**	**C**_****1****_		**C**_****2****_		**C**_****3****_	
**Sample**	**Larvae**	**Puparial cases**	**Larvae**	**Puparial cases**	**Larvae**	**Puparial cases**
Puparial cases	*N* = 16	–	*N* = 16	–	*N* = 17	–
	*Z = –*0.476		*Z = –*1.641		*Z = –*0.962	
	*p =* 0.678		*p =* 0.114		*p =* 0.376	
Adults	*N* = 16	*N* = 14	*N* = 17	*N* = 15	*N* = 17	*N* = 16
	*Z* = −2.276	*Z = –*0.064	*Z = –*2.694	*Z = –*0.347	*Z = –*2.983	*Z = –*2.836
	***p*** **=** **0.023**	*p =* 1.000	***p*** **=** **0.006**	*p =* 0.778	***p*** **=** **0.002**	***p=*** **0.004**

*Bold values denote statistical significance at the p <0.05*.

The mean bioaccumulation factor for Cd in larvae reached a value of about 0.20–0.25, and was slightly higher for samples reared on the C_1_ substrate (Figure 1, Supplementary Table 3). However, differences in bioaccumulation were found to be significant only between larvae reared on the C_1_ and C_3_ substrates (Table 4). For puparial cases, the mean bioaccumulation factor was lower, reaching values of 0.16–0.23, and the lowest values were obtained for adult flies (between 0.12 and 0.18) with no significant differences among substrates (Table 4, Supplementary Table 3).

**Table 4:** Difference of Cd bioaccumulation factor in *Lucilia sericata* larvae, puparial cases and adults from control and Cd treated groups (C_0_-C_3_).

**Sample**	**Larvae**			**Puparial cases**			**Adults**		
**Substrate**	**C_**0**_**	**C_**1**_**	**C_**2**_**	**C_**0**_**	**C_**1**_**	**C_**2**_**	**C_**0**_**	**C_**1**_**	**C_**2**_**
C_1_	*N* = 15	–	–	*N* = 14	–	–	*N* = 14	–	–
	*Z = –*2.946			*Z = –*0.447			*Z = –*1.469		
	***p=*** **0.001**			*p =* 0.703			*p =* 0.165		
C_2_	*N* = 15	*N* = 18	–	*N* = 15	*N* = 15	–	*N* = 15	*N* = 15	–
	*Z = –*2.239	*Z = –*1.987		*Z = –*0.579	*Z = –*0.231		*Z = –*2.430	*Z = –*0.347	
	***p=*** **0.027**	*p =* 0.051		*p =* 0.606	*p =* 0.871		***p=*** **0.015**	*p =* 0.775	
C_3_	*N* = 15	*N* = 18	*N* = 18	*N* = 16	*N* = 16	*N* = 17	*N* = 16	*N* = 16	*N* = 17
	*Z = –*1.414	*Z = –*2.075	*Z = –*0.662	*Z = –*0.265	*Z = –*0.265	*Z = –*0.289	*Z = –*2.805	*Z = –*1.323	*Z = –*1.732
	*p =* 0.183	***p=*** **0.041**	*p =* 0.546	*p =* 0.839	*p =* 0.848	*p =* 0.816	***p=*** **0.004**	*p =* 0.213	*p =* 0.094

*Bold values denote statistical significance at the p < 0.05*.

### Thallium Accumulation in Larvae, Puparial Cases, and Adults of *L. sericata*

**Figure 2:**
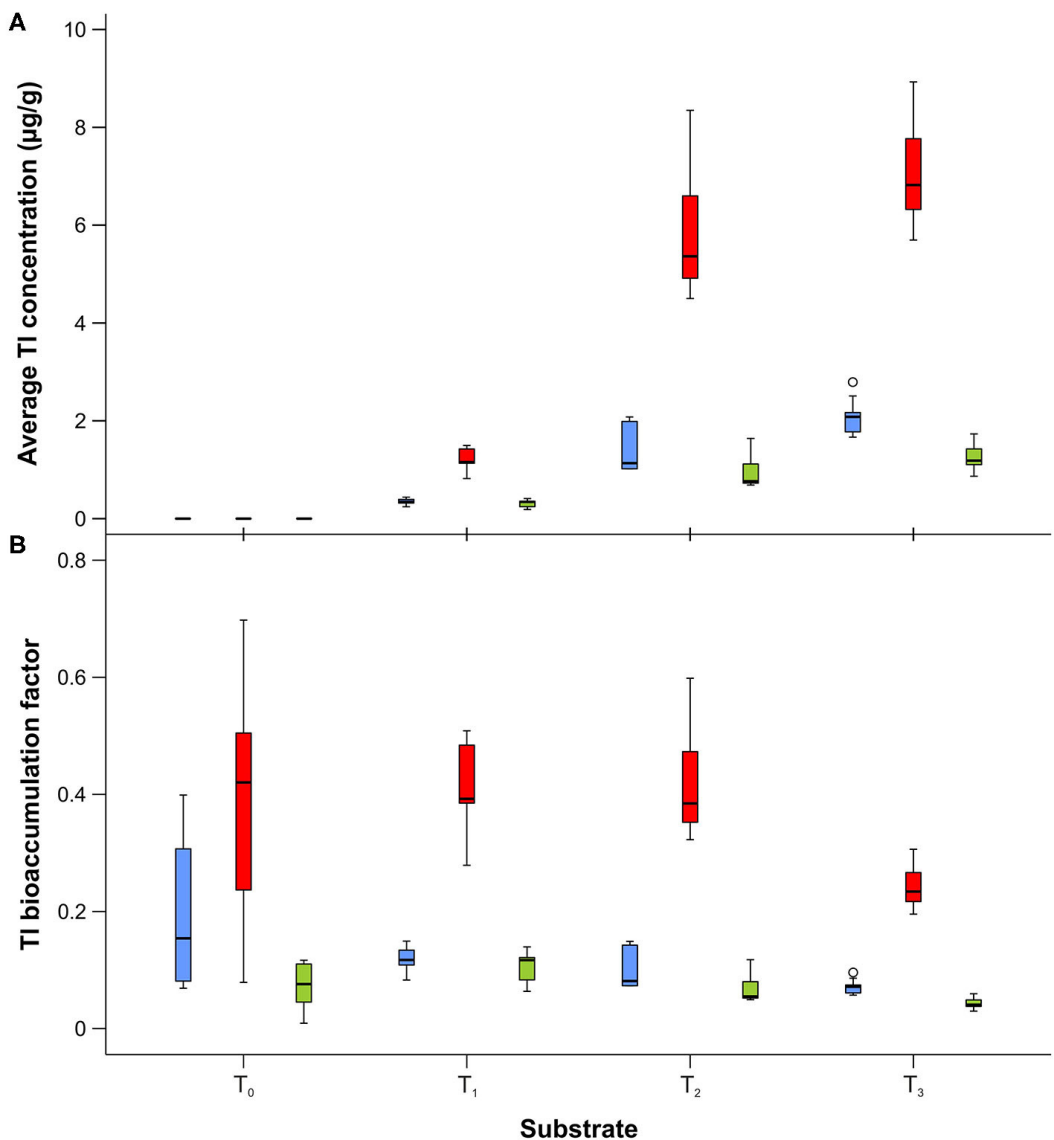
Box and whisker plots showing differences in average thallium concentration **(A)** and bioaccumulation factor **(B)** in larvae (blue), puparial cases (red), and adults (green) of *Lucilia sericata* exposed to different Tl concentration (T_0_-T_3_). Boxes indicate median and first and third quartiles. Whiskers represent the minimal and maximal values within 1.5 times the interquartile range. Open circles are outliers with values more than 1.5 times the interquartile range.

ICP-MS was used to determine the thallium content (as d.w.) in control (unenriched) liver tissues and liver samples enriched with different concentrations of metal. In control tissues (T_0_), the Tl concentration was 1.00 ± 0.75 ng g^−1^, while enriched liver samples had concentrations of 2.943 ± 0.071 μg g^−1^ in T_1_, 13.95 ± 0.15 μg g^−1^ in T_2_, and 29.1 ± 2.0 μg g^−1^ in T_3_. All substrates contained Tl concentrations that were below the lethal dose (LD_50_), which for different thallium salts has been reported to be in the range of 6–40 μg g^−1^ for humans and 29–39 μg g^−1^ for rats (Agency for Toxic Substances Disease Registry, 1999). The mean thallium concentrations in the developmental stages of *L. sericata* studied here are presented in Supplementary Table 2. The Tl content in larvae and adults ranged from 0.3 to 2.1 μg g^−1^, and in puparial cases from 1.2 to 7.0 μg g^−1^. Thallium concentrations generally increased in all stages—larvae, puparial cases, and adults—with an increase in the metal concentration in the food substrate (Figure 2, Table 5). Non-significant differences were only observed in larvae as well as puparial cases between T_2_ and T_3_ substrates (Table 5). The correlation between concentrations of Tl in the substrate and Tl levels in all developmental stages was not linear, and instead demonstrated a saturable relationship (Supplementary Figure 1). Thallium concentrations were significantly higher in puparial cases than in larvae and adult flies for all Tl-treated groups (Figure 2, Table 6). Tl concentrations also tended to be lower in adult flies compared to larvae, but this difference was only significant for the group reared on the substrate with the highest metal concentration, T_3_ (Table 6).

**Table 5:** Difference of Tl concentration determined in *Lucilia sericata* larvae, puparial cases and adults from control and Tl treated groups (T_0_-T_3_).

**Sample**	**Larvae**			**Puparial cases**			**Adults**		
**Substrate**	**T_**0**_**	**T_**1**_**	**T_**2**_**	**T_**0**_**	**T_**1**_**	**T_**2**_**	**T_**0**_**	**T_**1**_**	**T_**2**_**
T_1_	*N* = 10	–	–	*N* = 17	–	–	*N* = 17	–	–
	*Z = –*2.558			*Z = –*3.464			*Z = –*3.464		
	***p=*** **0.008**			***p<*** **0.001**			***p<*** **0.001**		
T_2_	*N* = 9	*N* = 11	–	*N* = 15	*N* = 16	–	*N* = 16	*N* = 17	–
	*Z = –*2.449	*Z = –*2.739		*Z = –*3.240	*Z = –*3.334		*Z = –*3.361	*Z = –*3.464	
	***p=*** **0.015**	***P=*** **0.005**		***p<*** **0.001**	***p<*** **0.001**		***p<*** **0.001**	***p<*** **0.001**	
T_3_	*N* = 13	*N* = 15	*N* = 14	*N* = 17	*N* = 18	*N* = 16	*N* = 17	*N* = 18	*N* = 17
	*Z = –*2.777	*Z = –*3.182	*Z = –*1.933	*Z = –*3.464	*Z = –*3.576	*Z = –*1.958	*Z = –*3.464	*Z = –*3.576	*Z = –*2.213
	***p=*** **0.003**	***p<*** **0.001**	*P =* 0.058	***p<*** **0.001**	***p<*** **0.001**	*P =* 0.055	***p<*** **0.001**	***p<*** **0.001**	***P=*** **0.026**

*Bold values denote statistical significance at the p < 0.05*.

**Table 6:** Difference of Tl concentration in *Lucilia sericata* larvae, puparial cases, and adults from groups treated with the same Tl concentration.

**Substrate**	**T**_****1****_		**T**_****2****_		**T**_****3****_	
**Sample**	**Larvae**	**Puparial cases**	**Larvae**	**Puparial cases**	**Larvae**	**Puparial cases**
Puparial cases	*N* = 15	–	*N* = 12	–	*N* = 18	–
	*Z = –*3.182		*Z = –*2.842		*Z = –*3.576	
	***p=*** **0.001**		***p=*** **0.003**		***p<*** **0.001**	
Adults	*N* = 15	*N* = 18	*N* = 13	*N* = 15	*N* = 18	*N* = 18
	*Z = –*0.943	*Z = –*3.576	*Z = –*1.757	*Z = –*3.240	*Z = –*3.400	*Z = –*3.576
	*p =* 0.394	***p<*** **0.001**	*p =* 0.096	***p<*** **0.001**	***p<*** **0.001**	***p<*** **0.001**

*Bold values denote statistical significance at the p < 0.05*.

The mean bioaccumulation factor for Tl was significantly higher in puparial cases (BAF in the range of 0.24–0.42) than in larvae (0.07–0.12) or adults (0.04–0.10) (Supplementary Table 3). BAF generally decreased with an increase in the metal concentration of the food substrate, and the biggest differences were observed for higher thallium concentrations (Figure 2, Table 7).

**Table 7:** Difference of Tl bioaccumulation factor in *Lucilia sericata* larvae, puparial cases, and adults from control and Tl treated groups (T_0_-T_3_).

**Sample**	**Larvae**			**Puparial cases**			**Adults**		
**Substrate**	**T_**0**_**	**T_**1**_**	**T_**2**_**	**T_**0**_**	**T_**1**_**	**T_**2**_**	**T_**0**_**	**T_**1**_**	**T_**2**_**
T_1_	*N* = 10	–	–	*N* = 17	–	–	*N* = 17	–	–
	*Z = –*0.213			*Z* = 0.000			*Z = –*1.925		
	*p =* 0.912			*p =* 1.000			*p =* 0.060		
T_2_	*N* = 9	*N* = 11	–	*N* = 15	*N* = 16	–	*N* = 16	*N* = 17	–
	*Z = –*0.735	*Z = –*0.913		*Z* = 0.000	*Z = –*0.053		*Z* = 0.000	*Z = –*2.502	
	*p =* 0.559	*p =* 0.425		*p =* 1.000	*p =* 1.000		*p =* 1.000	***p=*** **0.009**	
T_3_	*N* = 13	*N* = 15	*N* = 14	*N* = 17	*N* = 18	*N* = 16	*N* = 17	*N* = 18	*N* = 17
	*Z = –*1.852	*Z = –*2.946	*Z = –*1.933	*Z = –*1.732	*Z = –*3.311	*Z = –*3.334	*Z = –*1.828	*Z = –*3.576	*Z = –*2.983
	*p =* 0.075	***p=*** **0.001**	*p =* 0.060	*p =* 0.094	***p<*** **0.001**	***p<*** **0.001**	*p =* 0.070	***p<*** **0.001**	***p=*** **0.001**

*Bold values denote statistical significance at the p < 0.05*.

### Larval Weight and Puparial Size

**Figure 3:**
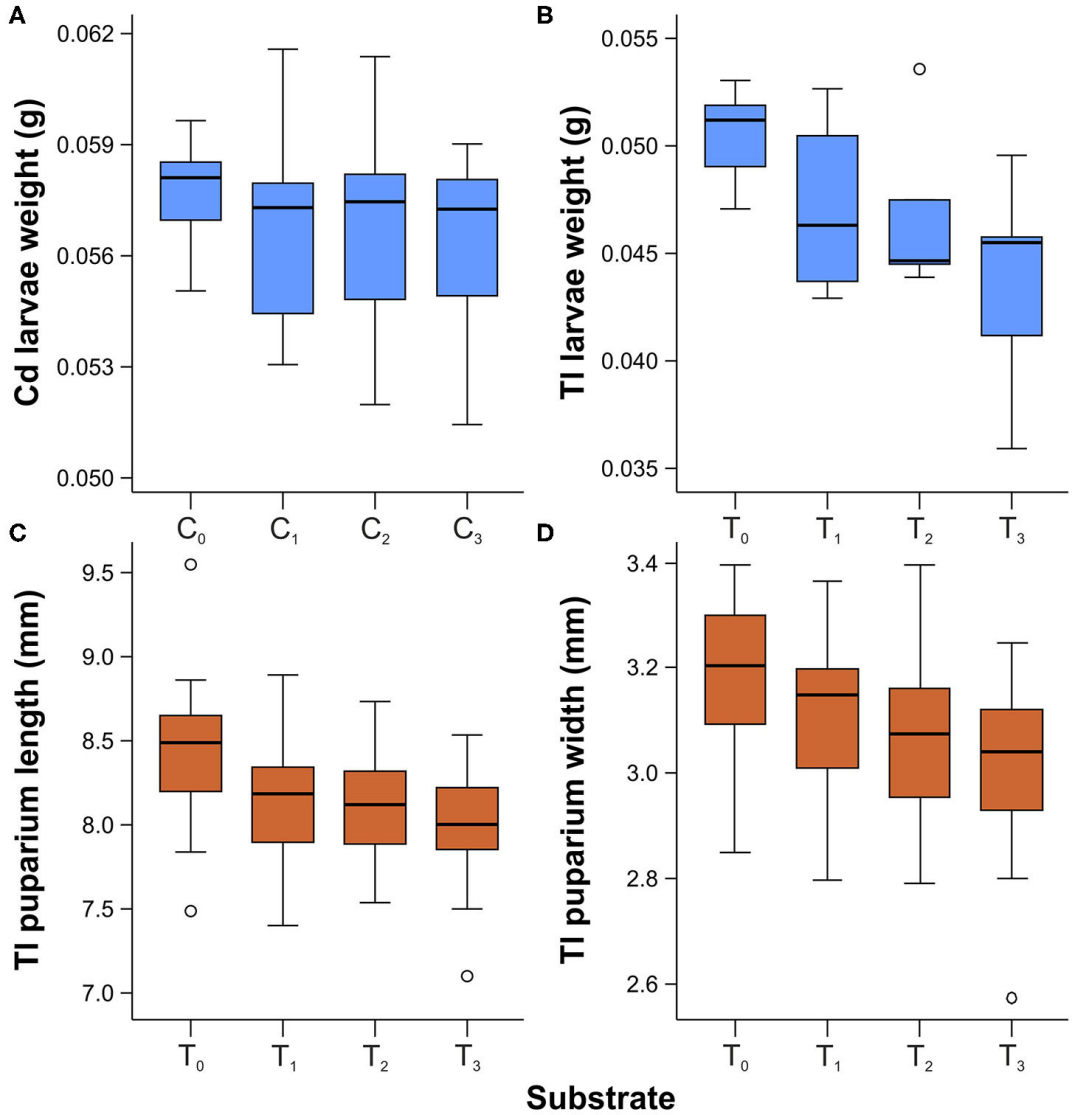
Box and whisker plots showing differences in weight of third instar larvae reared on substrates enriched with cadmium **(A)** and thallium **(B)**, and length **(C)** and width **(D)** of puparia acquired from larvae reared on substrate enriched with thallium. Boxes indicate median and first and third quartiles. Whiskers represent the minimal and maximal values within 1.5 times the interquartile range. Open circles are outliers with values more than 1.5 times the interquartile range.

The mean weight of third-instar larvae was generally lower for samples reared on substrates that contained cadmium or thallium (Figure 3). However, significant differences among groups were observed only for larvae reared with thallium [ANOVA: *F*_(3,22)_ = 4.125, *p* < 0.018], not with cadmium [ANOVA: *F*_(3,29)_ = 0.476, *p* = 0.701]. *Post-hoc* comparisons using the Tukey HSD test revealed that the mean weight of third-instar larvae was significantly lower when raised on the T_3_ substrate compared to the T_0_ substrate (*M* = 0.007, SD = 0.002, *p* = 0.010).

Significant differences were observed in the length [ANOVA: *F*_(3,116)_ = 7.818, *p* < 0.001] and width [ANOVA: *F*_(3,116)_ = 6.721, *p* < 0.001] of puparia acquired from larvae reared with different thallium concentrations (Figure 3). *Post-hoc* comparisons using the Tukey HSD test indicated that puparia from the T_0_ substrate were significantly longer than those from the T_1_ (*M* = 0.275, SD = 0.092, *p* = 0.017), T_2_ (*M* = 0.311, SD = 0.092, *p* = 0.006), and T_3_ (*M* = 0.431, SD = 0.092, *p* < 0.001) groups. Instead, puparia length did not differ significantly among the metal-enriched substrates (T_1_, T_2_, and T_3_). Similarly, a Tukey HSD test revealed that the mean width of puparia from the T_0_ substrate was significantly larger than values obtained from T_2_ (*M* = 0.108, SD = 0.038, *p* = 0.030) and T_3_ (*M* = 0.170, SD = 0.038, *p* < 0.001). The width of puparia did not differ significantly between samples from T_0_ and T_1_ or among samples from T_1_, T_2_, and T_3_ (*p* > 0.05).

## Discussion

### Accumulation of Cadmium and Thallium

To the best of our knowledge, this report presents the first examination of the relationship between Tl exposure and bioaccumulation in different developmental stages of the forensically important blowfly *L. sericata*. The effect of cadmium on the development and survival of blowfly populations has been studied for *C. albiceps* (Al-Misned, 2001, 2003), *C. megacephala* (Singh and Bhupinderjit, 2017), and *L. sericata* (Simkiss et al., 1993; Moe et al., 2001), but only in a few studies bioaccumulation factors for Cd have been calculated.

When we reared larvae of *L. sericata* on a metal-enriched substrate, we observed different accumulation patterns for cadmium and thallium. For Cd, there was clearly a linear relationship between the Cd content of the liver substrate and that found in different developmental stages of *L. sericata*, with correlation coefficients (*R*) in the range of 0.994–0.997. Instead, the relationship for Tl was obviously nonlinear, indicating that Tl accumulation is saturable at higher metal concentrations in all developmental stages (R between 0.998 and 1.000). In this, our results are similar to those of Belowitz et al. (2014), who reported saturable accumulation of Tl by larvae of *C. riparius*, with a maximum accumulation (*B*_max_) of ~4,600 μmol kg^−1^ wet mass. Those authors hypothesized that this phenomenon was the result of the limited availability of metal-binding proteins, such as metallothioneins (cysteine-rich proteins), that could decrease the metabolically available fraction. However, this hypothesis is not consistent with our results, as both Cd and Tl have the ability to bind sulfhydryl groups (–SH), and the responses of *L. sericata* to high doses of Cd and Tl were clearly different. Here, the bioaccumulation pattern for Cd was similar to that described for different developmental stages of the Asian Corn Borer, *Ostrinia furnacalis*, a type of grass moth (Luo et al., 2020). Our observation that the content of both Cd and Tl in adult flies was lower than in larvae was also consistent with previous reports (Kraus et al., 2014; Luo et al., 2020).

In this study, we noted clear differences between Cd and Tl with respect to accumulation in puparial cases (Figures 1, 2): mean Cd content was lower in puparial cases than in larvae, while the opposite was true for Tl. Our results thus provide evidence that, in this species, thallium is accumulated in the puparial cases (Figure 2). Previous studies have reported that one mechanism of detoxification among insects involves the exuviae, the coverings shed by the final larval stage or newly emerged adults. In the dragonfly species *Gomphus flavipes*, Simon et al. (2019) found that, for certain metals—Al, Fe, and Mn—and under certain living conditions, exuviae contained higher metal concentrations than either larvae or adults, demonstrating that molting plays a role in detoxification. Similarly, Kraus et al. (2014) reported that some trace elements, such as Zn, Cd, and Cu, were lost during metamorphosis, leading to 2- to 125-fold higher concentrations in larvae compared to adults, and a higher exposure risk for predators of larvae compared to predators of adults. Moreover, Muscatello and Liber (2009) found that between 22% and 58% of the U accumulated in *Chironomus tentans* was lost via the pupal skin during metamorphosis, which confirmed an important role of the pupal case in detoxification. Our finding that the Tl content in adults was lower than in larvae, and considerably lower than in puparial cases, suggests that this mechanism probably dominates in *L. sericata*'s detoxification of Tl. However, it should be considered that metals can be also excreted in the meconium of last-instar larvae or newly emerged adults. Indeed, Simon et al. (2019) reported that <10% of the metal burden lost during metamorphosis could be attributed to the exuviae whereas more than 50% was typically lost through meconium. This latter mechanism is more likely to play a role for Cd, as the concentration of Cd in adults was lower than in larvae, while concentrations in larvae and puparial cases were not statistically different. Furthermore, differences in metal concentration among different developmental stages of *L. sericata* were most visible in the groups reared on the substrate with the highest Cd concentration (Figure 1, Supplementary Table 2). These results are consistent with the work of Luo et al. (2020), who studied the routes of Cd excretion in *O. furnacalis* and found high levels of Cd in feces (95.49 μg g^−1^), silk (82.98 μg g^−1^), and pupal cases (76.87 μg g^−1^). Simkiss et al. (1993) observed an almost constant concentration of Cd in emerging adults of *L. sericata*, but the Cd concentration in puparial cases varied, from levels that were similar to those of adults to ones that were significantly higher.

To compare the transfer of toxins from the surrounding environment to a living organisms, the bioaccumulation factor is often used. Although cadmium is very readily assimilated, and its concentration in organisms is often higher than in their food (Moe et al., 2001), we observed that the mean BAF of Cd in larvae was about 0.20–0.25 (Figure 1; Supplementary Table 3), and was even lower for adult flies, reaching values between 0.12 and 0.18, with no significant differences among substrates. These data are comparable to the results obtained by Diener et al. (2015), who studied the bioaccumulation of Cd in different life stages of *Hermetia illucens* that were reared on chicken feed spiked with heavy metals. In that study, BAF values decreased from 0.21 to 0.13 with an increase in the concentration of Cd in feed from 2 to 50 μg g^−1^. For puparial cases, higher values were obtained (in the range of 0.86–1.41) than those found in our study. Instead, a species of ground beetle, *Poecilus cupreus*, that was fed Cd-contaminated larvae of *Musca domestica* demonstrated much lower BAF values, in the range of 0.001 to 0.046, depending on the accumulation period (Kramarz, 1999). Contradictory results were obtained by Moe et al. (2001), who observed that concentrations of Cd in adult flies of *L. sericata* (up to 400 μg g^−1^ dry mass) were up to eight times the wet-mass concentration in the diet. However, that study used a different compound of Cd (cadmium(II) acetate) and type of nutrient substrate (agar, yeast, and horse blood) than the present work. To date, the only report in the literature for thallium described the uptake of waterborne Tl by larvae of *C. riparius* (Belowitz et al., 2014). Concentration factors (CFs) that indicated how much more Tl was in insect tissue relative to the aqueous concentration were calculated. In that study, CFs of Tl in whole animals were 18- and 5-fold at the two lowest Tl exposures (0.7 and 5.5 μg mL^−1^), and ~4-fold over the range of the higher exposure concentrations (5.5–150 μg mL^−1^). In our study, the BAF values for Tl were always below 1, with the highest values observed for puparial cases.

To date, no information has been published regarding the potential use of puparial cases of *L. sericata* as toxicological evidence of metal poisoning. Moreover, the Guidelines for Collection of Biological Samples for Clinical and Forensic Toxicological Analysis (Dinis-Oliveira et al., 2016) only mention the use of fly larvae, and only for qualitative analysis. Here we demonstrate that empty puparial cases may also provide useful forensic information. For Tl, an increased content in insect remains could be an indicator of the high content of this metal in larval feed. However, as the bioaccumulation pattern is not linear, the results should only be considered qualitatively. For Cd, the concentration in puparial cases was proportional to the Cd content of the substrate, but the application of this relationship to quantitative analyses should be further studied for different Cd compounds over a wider range of concentrations. Because empty puparial cases can be found in the proximity of human remains even after many years, this type of information has the potential to be highly useful in interpreting the analytical results of legal and medical investigations of accidental and intentional poisonings.

### Effect of Cadmium and Thallium on Life History Traits of *L. sericata*

For the population of *L. sericata* tested here, the weight of larvae and size of puparia were affected by the presence of metal in the food substrate. In particular, we found that thallium had a negative effect on larval growth, with higher Tl concentrations resulting in lower weight and smaller puparial size. This is a novel finding in the forensic entomotoxicology literature, since to our knowledge thallium determination and its effect on fly development has not been previously assessed. Earlier investigations of Cd in *L. sericata* reported that the mean weight of both larvae and puparia decreased with increasing cadmium concentration in the food substrate (Simkiss et al., 1993; Singh and Bhupinderjit, 2017). Although our results did not reveal any statistically significant differences in larval weight between control and cadmium treatments, mean weight was slightly lower for larvae reared on cadmium compared to the control population. Various studies have reported a general reduction in insect growth as a result of exposure to cadmium (Schmidt et al., 1992; Ortel, 1996; Cervera et al., 2004; Sildanchandra and Crane, 2009) and other toxic metals (Purschke et al., 2017). However, Płachetka-Bozek et al. (2018) found that certain cadmium concentration in food had a variable effect on the growth of *Spodoptera exigua* (Lepidoptera: Noctuidae) larvae. These inconsistencies may arise from differences in the cadmium source used, the concentration in the food substrate, the type of feeding substrate, rearing conditions, or fly population tested (Simkiss et al., 1993; Silva et al., 2017; Płachetka-Bozek et al., 2018).

### Methodological Commentary

A common weakness of numerous experimental studies is lack of standardization in the methods used, which makes it difficult to replicate results (Chophi et al., 2019). In this work, we describe our methodology in detail with the goal of facilitating comparisons among the results of entomotoxicological studies.

We chose as a model organism the commonly available insect species *L. sericata*, belonging to the family of true flies (Diptera). To simulate the process of decay of the feeding substrate, as well as the ingestion and accumulation of toxins, a homogenized meat substrate spiked with cadmium or thallium was used to feed larvae. The use of liver matrix has been criticized in the past because certain toxicants (e.g., drugs) may undergo chemical reactions in liver tissues (Silva et al., 2017). However, in our view these shortcomings did not apply to enrichment with metals. In a pilot experiment, we optimized the method of homogenization of the metal-enriched substrate and confirmed the homogeneity of the solid substrate. In addition to generating a more homogenous substrate, the use of ultrasound for homogenization reduced the risk of sample loss or contamination from laboratory tools during substrate preparation. As recommended by the rules for collection of insects from the scene (Brundage and Byrd, 2016), we collected a fixed number of individuals (≥15) from each substrate; before being frozen for storage, larvae were rinsed with Milli-Q water to avoid external contamination from the metal-enriched substrate. We chose to not use an acidic solution for rinsing samples in order to avoid causing additional stress for the organisms. Moreover, Timmermans et al. (1992) reported no significant difference in Cd concentration for larvae of *C. riparius* that were rinsed in distilled water vs. those rinsed in diluted nitric acid.

The determination of Cd and Tl in forensic samples is a challenging task due to the generally limited amounts of sample available for analysis. Here, this problem was solved by using ICP-MS, as the calculated method detection limit allowed to obtain reliable results even for samples smaller than 0.01 g. The method limit of detection, that includes both the instrumental and procedural constituents, i.e., dilution and mineralization steps, should be preferably reported. The reproducibility of results of Cd and Tl determination in samples between parallel experiments was acceptable (below 25%), only in single cases slightly exceeded 30%. It must be mentioned that individual organisms were used for mineralization, without any initial grinding or homogenization of dried biomass. We expect that the homogenization of samples would further improve the reproducibility of results. Finally, the accuracy of our method was verified by analysis of certified reference materials.

## Conclusions

In this study, we demonstrate that larvae, adults, and empty puparial cases of *L. sericata* found on decomposing human remains, can be used as an alternative material for the detection of Cd and Tl present in the body at the time of death. Our experiment revealed that these metals accumulated in larvae feeding on contaminated liver substrates, and the ingested metals were retained through the puparial stage and were detected in the adult insects. The accumulation of Cd and Tl was studied for different metal concentration in the food substrate. It was observed that the metal content in larvae, puparial cases and adults exposed to contaminated liver substrate was significantly higher than those exposed to control substrate and increased with increasing metal concentration in the liver. Of the three developmental stages analyzed, the highest average content of Cd was found in larvae, whereas for Tl, the highest bioaccumulation factor was observed for puparial cases. The accumulation of thallium in these chitinized remnants could be of great importance in forensic examinations, as puparial cases can be found near human remains for an extremely long period of time. To the best of our knowledge, this is the first report on thallium accumulation in different developmental stages of a forensically important blowfly, and demonstrates the potential usefulness of this kind of evidence in entomotoxicological investigations. Our experiments also revealed that exposure to thallium had an adverse effect on larval growth, resulting in lower mean weight and smaller puparial size. Small quantity of samples, especially puparial cases, required the use of sensitive analytical method of Cd and Tl determination. This study confirmed that the ICP-MS method described here is characterized by low limits of detection and quantification, very good precision, acceptable reproducibility, and good accuracy, and was thus suitable for the determination of Cd and Tl in different developmental stages of blowfly.

## Data Availability Statement

The raw data supporting the conclusions of this article will be made available by the authors, without undue reservation.

## Author Contributions

JM investigation, data calculation, and writing original document. KD investigation, statistical analysis, and writing original manuscript. MT and JD investigation. BG-Ż conceptualization, writing, and supervision. All authors contributed to the article and approved the submitted version.

## Conflict of Interest

The authors declare that the research was conducted in the absence of any commercial or financial relationships that could be construed as a potential conflict of interest.

## References

[B1] Agency for Toxic Substances and Disease Registry (1992). Toxicological Profile for Thallium. U.S. Public Health Service. 38091458

[B2] Agency for Toxic Substances and Disease Registry (1999). Toxicological Profile for Cadmium. Atlanta, GA: U.S. Department of Health and Human Services, Public Health Service.

[B3] Al-Misned F. A. M. (2001). Biological effects of cadmium on life cycle parameters of *Chrysomya albiceps* (Wiedemann) (Diptera: Calliphoridae). Kuwait J. Sci. Eng. 28, 179–188.

[B4] Al-Misned F. A. M. (2003). Effect of cadmium on the longevity and fecundity of the blowfly *Chrysomya albiceps* (Wiedemann) (Diptera: Calliphoridae). Kuwait J. Sci. Eng. 30, 81–94.

[B5] Barwick V. J. Ellison S. L. R. (2000). VAM Project 3.2.1 Development and Harmonisation of Measurement Uncertainty Principles, Part (d): Protocol for Uncertainty Evaluation from Validation Data. LGC (Teddington) Limited.

[B6] Belowitz R. Leonard E. M. O'Donnell M. J. (2014). Effects of exposure to high concentrations of waterborne Tl on K and Tl concentrations in *Chironomus riparius* larvae. Comp. Biochem. Phys. C. 166, 59–64. 10.1016/j.cbpc.2014.07.003 25046737

[B7] Beyer J. Enos W. Stalic M. (1980). Drug identification through analysis of maggots. J. Forensic Sci. 25, 411–412. 10.1520/JFS12147J 7391801

[B8] Braga M. V. Pinto Z. T. Carvalho Queiroz M. M. Matsumoto N. Blomquist G. J. (2013). Cuticular hydrocarbons as a tool for the identification of insect species: puparial cases from Sarcophagidae. Acta Trop. 128, 479–485. 10.1016/j.actatropica.2013.07.014 23932943 PMC3839396

[B9] Brundage A. Byrd J. H. (2016). Forensic entomology in animal cruelty cases. Vet. Path., 53, 898–909. 10.1177/0300985816651683 27480760

[B10] Bugelli V. Campobasso C. P. Verhoff M. A. Amendt J. (2017b). Effects of different storage and measuring methods on larval length values for the blow flies (Diptera: Calliphoridae) *Lucilia sericata* and *Calliphora vicina*. Sci. Justice 57, 159–164. 10.1016/j.scijus.2016.10.008 28454623

[B11] Bugelli V. Papi L. Fornaro S. Stefanelli F. Chericoni S. Giusiani M.. (2017a). Entomotoxicology in burnt bodies: a case of maternal filicide-suicide by fire. Int. J. Legal Med. 133, 1299–1306. 10.1007/s00414-017-1628-0 28691148

[B12] Byrd J. H. Castner J. L. (2010). Insects of forensic importance, in Forensic Entomology: the Utility of Arthropods in Legal Investigations, eds. Byrd J. H. Castner J. L. (Boca Raton, FL: CRC Press), 39–126.

[B13] Campobasso C. P. Gherardi M. Caligara M. Sironi L. Introna F. (2004). Drug analysis in blowfly larvae and in human tissues: acomparative study. Int. J. Legal Med. 118, 210–214. 10.1007/s00414-004-0448-1 15106008

[B14] Cavanagh J. B. (1991). What have we learnt from Graham Frederick Young? Reflections on the mechanism of thallium neurotoxicity. Neuropath. Appl. Neuro. 17, 3–9. 10.1111/j.1365-2990.1991.tb00687.x 2057049

[B15] Cervera A. Maymó A. C. Sendra M. Martínez-Pardo R. Garcerá M. D. (2004). Cadmium effects on development and reproduction of *Oncopeltus fasciatus* (Heteroptera: Lygaeidae). J. Insect Physiol. 50, 737–749. 10.1016/j.jinsphys.2004.06.001 15288207

[B16] Chang Y. Wen J. Cai J. Xiao-Ying W. Yang L. Yd G. (2012). An investigation and pathological analysis of two fatal cases of cadmium poisoning. Forensic Sci. Int. 220, 1–3. 10.1016/j.forsciint.2012.01.032 22349354

[B17] Chophi R. Sharma S. Sharma S. Singh R. (2019). Forensic entomotoxicology: current concepts, trends and challenges. J. Forensic Legal. Med. 67, 28–36. 10.1016/j.jflm.2019.07.010 31398663

[B18] Desenclos J. C. Wilder M. H. Coppenger G. W. Sherin K. Tiller R. VanHook R. M. (1992). Thallium poisoning: an outbreak in Florida, 1988. South. Med. J. 85, 1203–1206. 10.1097/00007611-199212000-00012 1470964

[B19] Diener S. Zurbrügg C. Tockner K. (2015). Bioaccumulation of heavy metals in the black soldier fly, *Hermetia illucens* and effects on its life cycle. J. Insects as Food Feed. 1, 261–270. 10.3920/JIFF2015.0030

[B20] Dinis-Oliveira R. J. Vieira D. N. Magalhães T. (2016). Guidelines for collection of biological samples for clinical and forensic toxicological analysis. Forensic Sci. Res. 1, 42–51. 10.1080/20961790.2016.1271098 30483610 PMC6197137

[B21] Gallagher M. B. Sandhu S. Kimsey R. (2010). Variation in developmental time for geographically distinct populations of the common green bottle fly, *Lucilia sericata* (Meigen). J. Forensic Sci. 55, 438–443. 10.1111/j.1556-4029.2009.01285.x 20102471

[B22] Gaudry E. Myskowiak J.-B. Chauvet B. Pasquerault T. Lefebvre F. Malgorn Y. (2001). Activity of the forensic entomology department of the French Gendarmerie. Forensic Sci. Int. 120, 68–71. 10.1016/S0379-0738(01)00427-3 11457612

[B23] Goff M. L. Miller M. L. Paulson J. D. Lord W. D. Richards E. Omori A. I. (1997). Effects of 3,4-methylenedioxymethamphetamine in decomposing tissues on the development of *Parasarcophaga ruficornis* (Diptera:Sarcophagidae) and detection of the drug in postmortem blood, liver tissue, larvae, and puparia. J. Forensic Sci. 42, 276–280. 10.1520/JFS14110J 9068186

[B24] Greenberg B. (1991). Flies as forensic indicators. J. Med. Entomol. 28, 565–577. 10.1093/jmedent/28.5.565 1941921

[B25] Hoffman R. S. Hoffman R. (2000). Thallium poisoning during pregnancy: a case report and comprehensive literature review. J. Toxicol. Clin. Toxicol. 38, 767–775. 10.1081/CLT-100102390 11192464

[B26] Karbowska B. (2016). Presence of thallium in the environment: sources of contaminations, distribution and monitoring methods. Environ. Monit. Assess. 188, 640. 10.1007/s10661-016-5647-y 27783348 PMC5080298

[B27] Kramarz P. (1999). Dynamics of accumulation and decontamination of cadmium and zinc in carnivorous invertebrates. 1. The ground beetle, *Poecilus cupreus* L. B. Environ. Contam. Toxiocol. 63, 531–537. 10.1007/s001289901013 10501733

[B28] Kraus J. M. Walters D. M. Wesner J. S. Stricker C. A. Schmidt T. S. Zuelling R. E. (2014). Metamorphosis alters contaminants and chemical tracers in insects: Implications for food webs. Environ. Sci. Technol. 48, 10957–10965. 10.1021/es502970b 25136925

[B29] Lech T. Sadlik J. K. (2017). Cadmium concentration in human autopsy tissues. Biol. Trace Elem. Res. 179, 172–177. 10.1007/s12011-017-0959-5 28220387

[B30] Luo M. Cao H.-M. Fan Y.-Y. Zhou X.-C. Chen J.-X. Chung H.. (2020). Bioaccumulation of cadmium affects development, mating behavior, and fecundity in the Asian Corn Borer, Ostrinia furnacalis. Insects. 11:7. 10.3390/insects11010007 31861761 PMC7022320

[B31] Meggs W. J. Hoffman R. S. Shih R. D. Weismann R. S. Goldfrank L. R. (1994). Thallium poisoning from maliciously contaminated food. J. Toxicol. Clin. Toxicol. 32, 723–730. 10.3109/15563659409017979 7966530

[B32] Miller M. Lord W. Goff M. Donnelly B. McDonough E. Alexis J. (1994). Isolation of Amitriptyline and Nortriptyline from fly puparia (Phoridae) and beetle exuviae (Dermestidae) associated with mummified human remains. J. Forensic Sci. 39, 1305–1313. 10.1520/JFS13717J

[B33] Moe S. J. Stenseth N. C. Smith R. H. (2001). Effects of a toxicant on population growth rates: sublethal and delayed responses in blowfly populations. Funct. Ecol. 15, 712–721. 10.1046/j.0269-8463.2001.00575.x

[B34] Muscatello J. R. Liber K. (2009). Accumulation and chronic toxicity of uranium over different life stages of the aquatic invertebrate *Chironomus tentans*. Arch. Environ. Contam. Toxicol. 57, 531–539. 10.1007/s00244-009-9283-1 19148695

[B35] Nishijo M. Nakagawa H. Suwazono Y. Nogawa K. Kido T. (2017). Causes of death in patients with Itaiitai disease suffering from severe chronic cadmium poisoning: a nested case-control analysis of a follow-up study in Japan. BMJ Open 7:e015694 10.1136/bmjopen-2016-015694 PMC5734474 28710217

[B36] Nolte K. B. Pinder R. D. Lord W. D. (1992). Insect larvae used to detect cocaine poisoning in a decomposed body. J. Forensic Sci. 37, 1179–1185. 10.1520/JFS13304J 1506834

[B37] Ortel J. (1996). Metal-supplemented diets alter carbohydrate levels in tissue and hemolymph of gypsy moth larvae (Lymantria dispar, Lymantriidae, Lepidoptera). Environ. Toxicol. Chem. 15, 1171–1176. 10.1002/etc.5620150723

[B38] Owings C. G. Banerjee A. Asher T. M. D. Gilhooly W. P. III. Tuceryan A. Huffine M.. (2019). Female blow flies as vertebrate resource indicators. Sci. Rep. 9:10594. 10.1038/s41598-019-46758-9 31332240 PMC6646386

[B39] Płachetka-Bozek A. Kafel A. Augustyniak M. (2018). Reproduction and development of Spodoptera exigua from cadmium and control strains under differentiated cadmium stress. Ecotoxicol. Environ. Saf. 166, 138–145. 10.1016/j.ecoenv.2018.09.016 30265877

[B40] Pounder D. J. (1991). Forensic entomo-toxicology. J. Forensic Sci. Soc. 31, 469–472. 10.1016/S0015-7368(91)73189-7 1797976

[B41] Powers R. H. Dean D. E. (2015). Forensic Toxicology: Mechanisms and Pathology. Boca Raton, FL: CRC Press.

[B42] Prado e Castro C. Serrano A. Martins da Silva P. Garcia M. D. (2012). Carrion flies of forensic interest: a study of seasonal community composition and succession in Lisbon, Portugal. Med. Vet. Entomol. 26, 417–431. 10.1111/j.1365-2915.2012.01031.x 22765479

[B43] Purschke B. Scheibelberger R. Axmann S. Adler A. Jäger H. (2017). Impact of substrate contamination with mycotoxins, heavy metals and pesticides on the growth performance and composition of black soldier fly larvae (*Hermetia illucens*) for use in the feed and food value chain. Food Addit. Contam. Part A. 34, 1410–1420. 10.1080/19440049.2017.1299946 28278126

[B44] Rafati Rahimzadeh M. Rafati Rahimzadeh M. Kazemi S. Moghadamnia A. A. (2017). Cadmium toxicity and treatment: an update. Caspian J. Intern. Med. 8, 135–145. 10.22088/cjim.8.3.135 28932363 PMC5596182

[B45] Reibe S. Doetinchem P. Madea B. (2010). A new simulation-based model for calculating post-mortem intervals using developmental data for *Lucilia sericata* (Dipt.: Calliphoridae). Parasitol. Res. 107, 9–16. 10.1007/s00436-010-1879-x 20440626

[B46] Reith S. (2009). Toxicological Review of Thallium and Compounds. Washington, DC: U.S. Environmental Protection Agency.

[B47] Sakai K. (2015). Routine Soil Analysis Using an Agilent 8800 ICP-QQQ. Application note. Agilent Technologies.

[B48] Schmidt G. H. Ibrahim N. M. M. Abdallah M. D. (1992). Long-term effects of heavy metals in food on developmental stages of *Aiolopus thalassinus* (Saltatoria: Acrididae). Arch. Environ. Con. Tox. 23, 375–382. 10.1007/BF00216248 1456784

[B49] Sigel A. Sigel H. Sigel R. K. O. (2013). Cadmium: From Toxicity to Essentiality. New York, NY: Springer.

[B50] Sildanchandra W. Crane M. (2009). Influence of sexual dimorphism in *Chironomus riparius* Meigen on toxic effects of cadmium. Environ. Toxicol. Chem. 19, 2309–2313. 10.1002/etc.5620190921

[B51] Silva E. I. T. Wilhelmi B. Villet M. H. (2017). Forensic entomotoxicology revisited - towards professional standardisation of study designs. Int. J. Leg. Med. 131, 1399–1412. 10.1007/s00414-017-1603-9 28567525

[B52] Simkiss K. Daniels S. Smith R. H. (1993). Effects of population density and cadmium toxicity on growth and survival of blowflies. Environ. Pollut. 81, 41–45. 10.1016/0269-7491(93)90026-K 15091835

[B53] Simon E. Tóthmérész B. Kis O. Jakab T. Szalay P. E. Vincze A. (2019). Environmental-friendly contamination assessment of habitats based on the trace element content of dragonfly exuviae. Water 11, 2200 10.3390/w11112200

[B54] Singh D. Bhupinderjit K. H. (2017). Effect of cadmium chloride on the development of *Chrysomya megacephala* (Diptera:Calliphoridae) and its importance to postmortem interval estimate. J. Forensic Sci. Criminal Invest. 3, 555622 10.19080/JFSCI.2017.03.555622

[B55] Timmermans K. R. Peeters W. Tonkes M. (1992). Cadmium, zinc, lead and copper in *Chironomus riparius* (Meigen) larvae (Diptera, Chironomidae): uptake and effects. Hydrobiologia. 241, 119–134. 10.1007/BF00008264

[B56] Tracqui A. Keyser-Tracqui C. Kintz P. Ludes B. (2004). Entomotoxicology for the forensic toxicologist: much ado about nothing? Int. J. Legal Med. 118, 194–196. 10.1007/s00414-004-0442-7 15164211

[B57] Wang J. Li Z. Chen Y. Chen Q. Yin X. (2008). The succession and development of insects on pig carcasses and their significances in estimating PMI in south China. Forensic Sci. Int. 179, 11–18. 10.1016/j.forsciint.2008.04.014 18534797

[B58] Zhu G.-H. Jia Z.-J. Yu X.-J. Wu K.-S. Chen L.-S. Lv J.-Y.. (2017). Predictable weathering of puparial hydrocarbons of necrophagous flies for determining the postmortem interval: a field experiment using Chrysomya rufifacies. Int. J. Legal Med. 131, 885–894. 10.1007/s00414-016-1507-0 28058571

