# Supplementary material for: Puparial Cases as Toxicological Indicators: Bioaccumulation of Cadmium and Thallium in the Forensically Important Blowfly Lucilia sericata
Source: Front Chem. 2020 Nov 17;8:586067. doi: 10.3389/fchem.2020.586067 (PMC7716273; doi:10.3389/fchem.2020.586067)
Supplement: Supplementary file 1 [file Data_Sheet_1.docx]

**Supplementary material**

**Title: Puparial cases as toxicological indicators: Bioaccumulation of cadmium and thallium in the forensically important blowfly *Lucilia sericata***

**Journal: Frontiers in Chemistry**

**DOI: 10.3389/fchem.2020.586067**

*Julita Malejko^a^, Krzysztof Deoniziak^b*^, Marlena Tomczuk^a^, Joanna Długokencka^a^, Beata Godlewska-Żyłkiewicz^a*^*

*^a^ Department of Analytical Chemistry, Faculty of Chemistry, University of Bialystok, Ciołkowskiego 1K, 15-245 Białystok, Poland;*

*^b^ Laboratory of Insect Evolutionary Biology and Ecology, Faculty of Biology, University of Bialystok, Ciołkowskiego 1J, 15-245 Białystok, Poland*

** Corresponding authors (krzysztofdeo@gmail.com, bgodlew@uwb.edu.pl)*


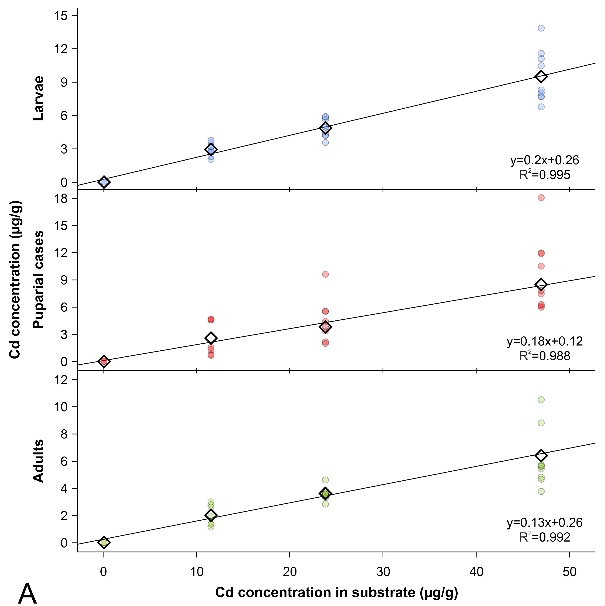

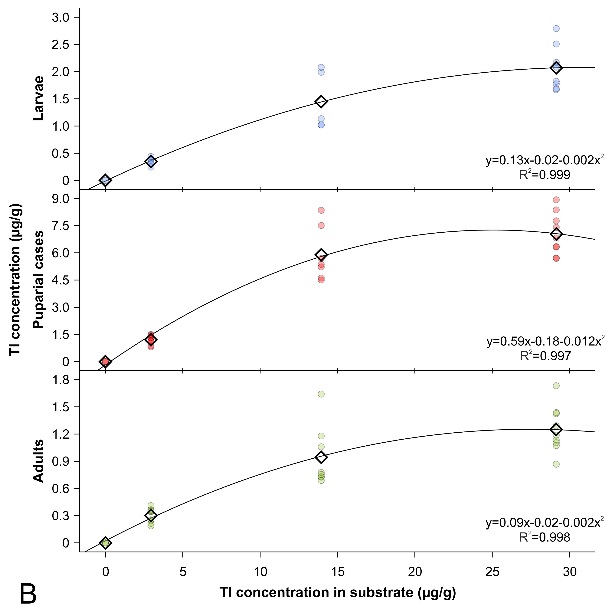


Supplementary Figure 1. Concentration of cadmium (A) and thallium (B) in studied developmental stages of *Lucilia sericata* in relation to metal concentration in feeding substrate. Diamonds indicate mean values, while circles measurements of individual samples. Trend lines represent a linear (A) and quadric (B) relationship between mean concentration of metal in different developmental stages of *Lucilia sericata* in relation to metal concentration in feeding substrate.

Supplementary Table 1. Agilent 8800 ICP-QQQ operating conditions.

| RF power | 1550 W |
| --- | --- |
| Plasma gas flow rate | 15 L min^-1^ |
| Auxiliary gas flow rate | 0.90 L min^-1^ |
| Carrier gas flow rate | 1.07 L min^-1^ |
| Sample depth | 10 mm |
| Sample inlet flow | 0.35 mL min^-1^ |
| Monitoredisotope  (integrationtime) | ^111^Cd (1.0 s), ^114^Cd (1.0 s),  ^205^Tl (0.3 s), ^115^In (0.3 s) |
| Plasmamode | General purpose |
| Scan type | Single Quad |
| Cell gasmode | No gas, He (5 mL min^-1^ flow rate) |

Supplementary Table 2. Concentration of cadmium and thallium (mean±SD) in samples of liver substrate, larvae, puparial cases and adults.

|  | Substrate | 0 | 1 | 2 | 3 |
| --- | --- | --- | --- | --- | --- |
|  | Sample | Mean ± SD  (ng g^-1^d.w.) | Mean ± SD  (µg g^-1^d.w.) | Mean ± SD  (µg g^-1^d.w.) | Mean ± SD  (µg g^-1^d.w.) |
| Cd | Liver | 94.1 ± 5.9 | 11.566 ± 0.028 | 23.83 ± 0.54 | 46.98 ± 0.56 |
|  | Larvae | 15.4±2.6 | 2.95± 0.56 | 4.89± 0.82 | 9.5 ± 2.4 |
|  | Puparial cases | 19.9± 6.7 | 2.6 ± 1.9 | 3.8 ± 1.4 | 8.5 ± 2.6 |
|  | Adults | 21.1± 5.1 | 2.04± 0.77 | 3.63 ± 0.50 | 5.6± 1.5 |
| Tl | Liver | 1.00 ± 0.75 | 2.943± 0.071 | 13.95± 0.15 | 29.1 ± 2.0 |
|  | Larvae | 0.19 ± 0.15 | 0.348± 0.068 | 1.45± 0.54 | 2.07± 0.38 |
|  | Puparial cases | 0.39 ± 0.21 | 1.20 ± 0.25 | 5.9 ± 1.5 | 7.0 ± 1.2 |
|  | Adults | 0.074± 0.040 | 0.303± 0.078 | 0.94 ± 0.33 | 1.25 ± 0.25 |

Supplementary Table 3. Bioaccumulation factor of cadmium and thallium (mean±SD) in larvae, puparial cases and adults.

|  | Substrate | 0 | 1 | 2 | 3 |
| --- | --- | --- | --- | --- | --- |
|  | Sample | Mean ± SD | Mean ± SD | Mean ± SD | Mean ± SD |
| Cd | Larvae | 0.164± 0.028 | 0.255 ± 0.049  0.225 ± 0.166  0.176 ± 0.066 | 0.205 ± 0.034  0.162 ± 0.061  0.152 ± 0.021 | 0.202 ± 0.050  0.181 ± 0.055  0.119 ± 0.031 |
|  | Puparial cases | 0.212± 0.071 |  |  |  |
|  | Adults | 0.225± 0.055 |  |  |  |
| Tl | Larvae | 0.194 ± 0.151 | 0.118 ± 0.023  0.409 ± 0.084  0.103 ± 0.026 | 0.104 ± 0.038  0.422 ± 0.106  0.068 ± 0.024 | 0.071 ± 0.013  0.242 ± 0.039  0.043 ± 0.009 |
|  | Puparial cases | 0.388± 0.207 |  |  |  |
|  | Adults | 0.074± 0.040 |  |  |  |
